# Case Report: Cutaneous T-cell lymphoma associated with biologic therapy: three cases and a literature review

**DOI:** 10.3389/fmed.2025.1544912

**Published:** 2025-04-28

**Authors:** Tingting Li, Guanyu Wang, Chunlei Zhang, Wenhui Wang, Chunting Li, Yimeng Wang

**Affiliations:** ^1^Department of Dermatology, Peking University Third Hospital, Beijing, China; ^2^Department of Dermatology, Tianjin People's Hospital, Tianjin, China

**Keywords:** cutaneous T-cell lymphoma, biologics, atopic dermatitis, dupilumab, JAK inhibitor

## Abstract

Cutaneous T-cell lymphoma (CTCL) often presents with early-stage clinical features indistinguishable from atopic dermatitis (AD), posing significant diagnostic challenges. Recent studies have highlighted cases where patients initially diagnosed with AD and treated with biologic agents were subsequently reclassified as having CTCL, though the nature of this relationship remains poorly understood. In this report, we present two cases of mycosis fungoides and one case of Sézary syndrome (SS), all initially diagnosed as AD and treated with biologic agents, including dupilumab. Furthermore, we conducted a literature review exploring potential associations between AD, biologic agents, and CTCL. Our objective is to improve clinicians’ ability to differentiate between AD and CTCL and to provide evidence supporting a possible association between biologic treatments and CTCL. Our findings highlight the need for heightened clinical vigilance and the routine use of skin biopsy in refractory eczematous presentations.

## 1 Introduction

Cutaneous T-cell lymphoma (CTCL) is a rare subtype of non-Hodgkin lymphoma that primarily involves T cells and predominantly affects the skin. The two main subtypes of CTCL are mycosis fungoides (MF) and Sézary syndrome (SS) (1). The clinical presentation of CTCL is highly variable, often mimicking other dermatological conditions, particularly atopic dermatitis (AD). AD is an inflammatory spongiform dermatitis characterized by a pruritic, scaly rash that often presents as erythema and plaques. Similarly, the common clinical features of MF include scaly erythema or plaques that closely mimic severe AD (2). This resemblance frequently leads to misdiagnosis, as the early manifestations of CTCL share significant overlap with AD in terms of symptoms and histopathological features. AD is a chronic, T-cell-mediated inflammatory skin disease commonly treated with biologic agents, which have revolutionized its management. However, recent studies have raised concerns about a potential association between biologic agents and CTCL (3). This relationship remains poorly understood, with possible explanations including misdiagnosis of CTCL as AD, or the possibility that biologic agents used to treat AD may contribute to the development or progression of CTCL.

In this study, we report three cases—two of MF and one of SS—that were initially diagnosed as AD. All three patients received biologic agents as part of their treatment for AD, including dupilumab, a widely used IL-4Rα inhibitor. We also conducted a comprehensive literature review to investigate the potential association between biologic agents and CTCL. Our aim is to enhance clinicians’ ability to differentiate between AD and CTCL, provide evidence for a possible association between biologic agents and CTCL, and offer recommendations for improving diagnostic accuracy and patient outcomes.

## 2 Case description

### 2.1 Case 1

**Figure 1:**
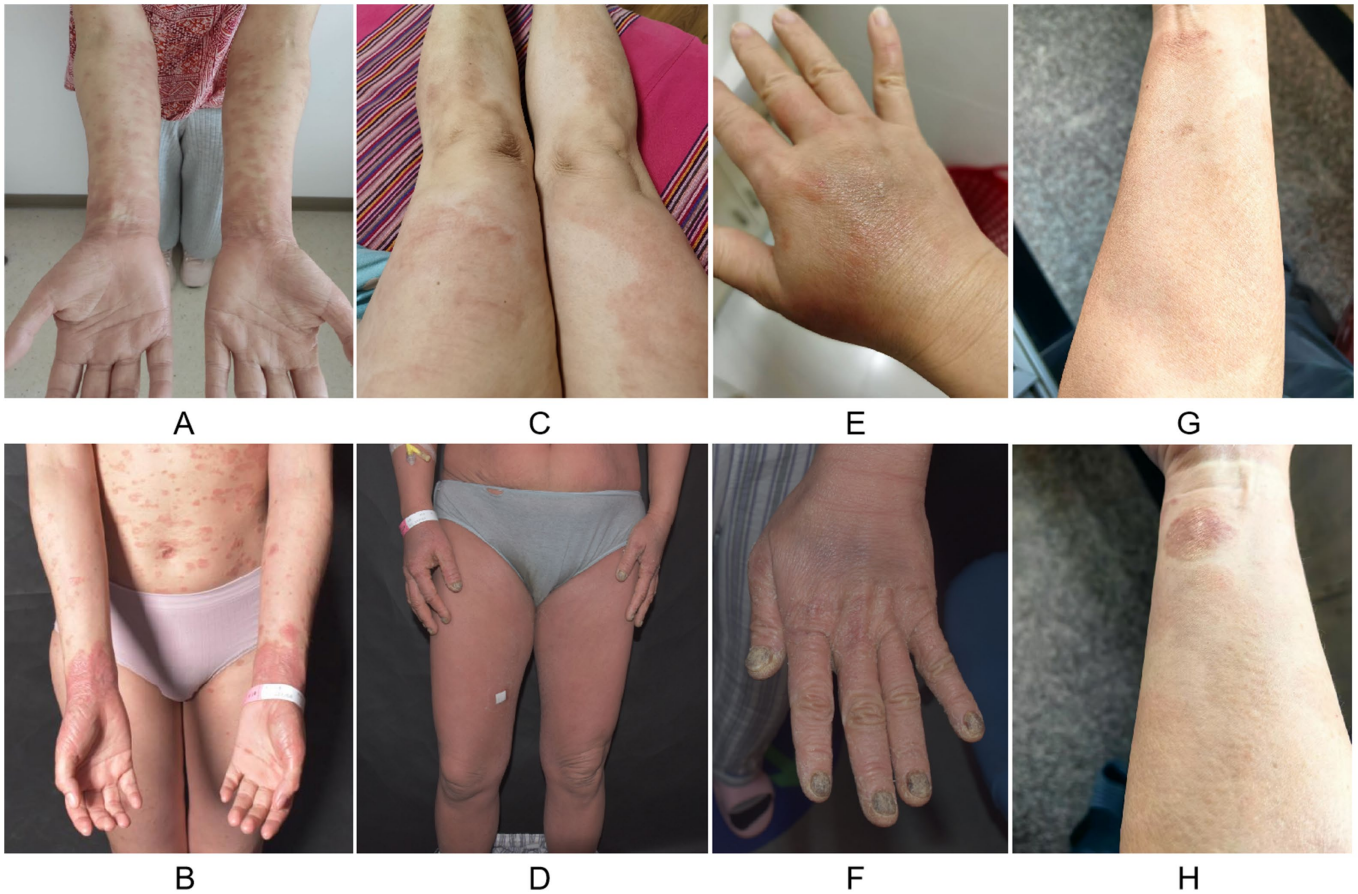
Clinical manifestations of cutaneous T-cell lymphoma in patients with atopic dermatitis before and after treatment with biologic agents: **(A)** Case 1: erythema on the limbs, covered with fine white scales; **(B)** Case 1: erythema and plaques on the face, neck, trunk, and limbs, covered with fine white scales; hands and wrists exhibit well-demarcated red plaques with hyperkeratosis; **(C)** Case 2: infiltrative, well-demarcated erythema on the lower extremities, covered with scales; **(D)** Case 2: generalized erythema on the face, trunk and limbs, with hyperkeratosis of the hands and feet, and feet exhibit the erythema base of the fusion of the pustules, BSA > 90%; **(E)** Case 2: normal nail appearance; **(F)** Case 2: all 20 nails appear turbid and yellowed, with surface roughness; **(G)** Case 3: erythema on the limbs, covered with fine white scales; and **(H)** Case 3: limbs can be seen with a clear boundary of the infiltrating dark erythematous plaques; the limbs have scattered hyperpigmented spots.

**Figure 2:**
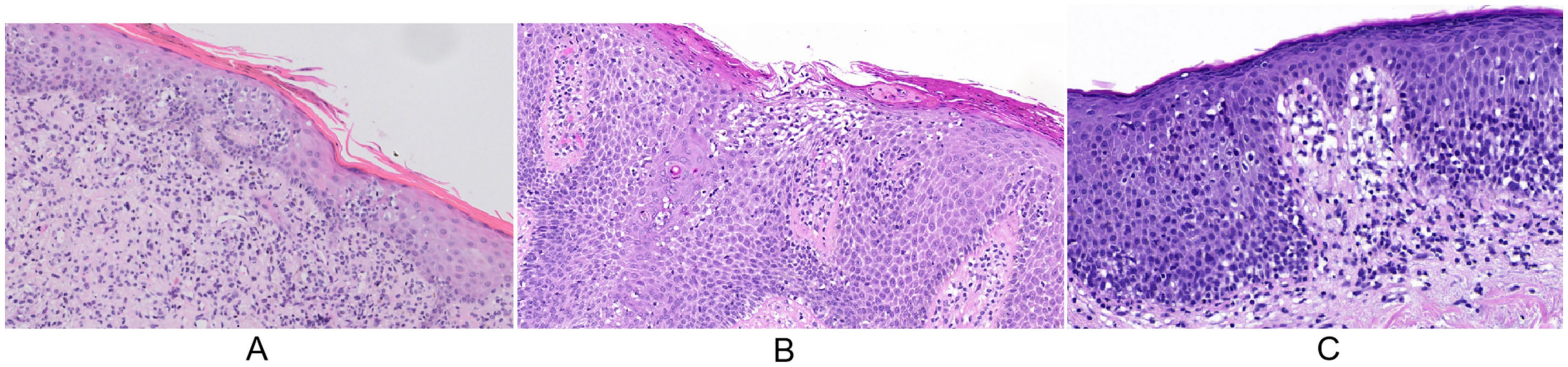
Pathological manifestations of cutaneous T-cell lymphoma in patients with atopic dermatitis treated with biologic agents: **(A)** Case 1: focal keratosis pilaris with mild epidermal hyperplasia, increased infiltration of single-nucleated cells into the epidermis, exhibiting perinuclear clear halos; Pautrier’s microabscesses and nuclear filamentous schizonts are present, and the entire dermis is infiltrated with mossy single-nucleated cells; **(B)** Case 2: focal keratosis pilaris and epidermal psoriasis-like irregular hyperplasia, infiltration of numerous single-nucleated cells into the epidermis, with prominent perinuclear accompanied by vacuoles; some cells exhibit deeply stained, irregularly shaped large nuclei, and Pautrier’s microabscesses and nuclear filamentous schizonts are visible; **(C)** Case 3 focal keratoconjunctivitis with superficial pustular plasma crusts; the epidermis shows mild to moderate irregular hyperplasia, visible intercellular edema, vacuolar changes in basal cells, the superficial layer of the dermis is infiltrated by mossy-like single-nucleated cells, with visible pro-epithelial cells, some of which are slightly oversized and heterogeneous, with isolated nuclear filamentous divisions (HE staining, 20×).

A 39-year-old woman presented to the hospital on 22 June 2021 with a 5-year history of recurrent erythema and papules accompanied by pruritus. Based on the Williams criteria, which include pruritus, involvement of flexural areas, generalized xerosis, and flexural eczema, she was diagnosed with AD. The disease severity scores for the patient were as follows: SCORing Atopic Dermatitis (SCORAD): 45, Eczema Area and Severity Index (EASI): 18.5, Peak Pruritus Numeric Rating Scale (PP-NRS): 8, Dermatology Life Quality Index (DLQI): 19, Patient-Oriented Eczema Measure (POEM): 17. Over the course of her treatment, the patient received 12 doses of dupilumab, 2 mg/day of baricitinib for 2 months, and 15 mg/day of upadacitinib for 1 month. During treatment, she developed new erythema and plaques, with the rash spreading further. The patient denied any history of allergic rhinitis or asthma. A specialist examination revealed erythema and plaques on the face, neck, trunk, and extremities, covered with white scales. Well-demarcated red plaques with hyperkeratosis were observed on the hands and wrists (Figures 1A,B). Laboratory tests revealed an IgE level of 1,010 U/L, while the remaining results were within normal limits. Skin biopsy revealed hyperkeratosis, epidermal hyperplasia, and mononuclear cell infiltration into the epidermis, with the presence of Pautrier’s microabscesses and mitosis. Lichenoid mononuclear infiltration involved the entire dermis (Figure 2A). Immunohistochemical analysis showed CD2 (+), CD3 (+), CD4 (+), CD5 (+), CD7 (partial+), CD8 (−), CD30 (10%+), TCRG, T-cell receptor gamma-A (+), and TCRG-B (+). PET/CT scans revealed multiple hypermetabolic lymph nodes in the right occipital, bilateral preauricular, bilateral parotid, bilateral cervical, left supraclavicular, and bilateral axillary regions. A lymph node biopsy confirmed lymphoma. The patient was diagnosed with MF (T2N1M0B0, stage IIA) and was treated with acitretin (30 mg/day), methotrexate (10 mg/week), narrowband ultraviolet B (NB-UVB) phototherapy, and topical corticosteroids. A 3-year follow-up showed significant improvement in her skin lesions and overall disease condition.

### 2.2 Case 2

A 40-year-old woman presented on 15 August 2023 with a 2-year history of recurrent rashes and pruritus affecting the trunk and extremities. She was diagnosed with AD based on the Williams criteria. The disease severity scores for the patient were as follows: SCORAD: 78, EASI: 47.4, PP-NRS: 10, DLQI: 25, POEM: 28. After receiving two doses of dupilumab, she developed erythema and pustules, prompting a switch to upadacitinib (15 mg/day for 1 month). Despite this change, her condition did not improve. The patient had a history of allergic rhinitis. A specialist examination revealed generalized erythema on the face, trunk, and extremities; hyperkeratosis of the hands and feet; confluent pustules on erythematous bases on both feet with partial ulceration and scaling; and involvement of all 20 nails, which were cloudy, yellow, and rough in texture. Body surface area involvement exceeded 90% (Figures 1C–F). Laboratory tests showed LDH: 677 U/L, β2-MG: 2.72 U/L, CD4/CD8 ratio: 19.43, and IgE: >2,500 IU/mL. EBV-DNA and EBV-IgM were negative. Skin pathology indicated focal hyperkeratosis, psoriasiform irregular hyperplasia, mononuclear cell infiltration into the epidermis, Pautrier’s microabscesses, and mitosis (Figure 2B). Immunohistochemical analysis revealed CD2 (+), CD3 (+), CD4 (+), CD5 (+), CD7 (<10%), CD8 (markedly reduced), CD30 (positive in a few cells), TCRB-A (+), and TCRG-A (+). PET/CT revealed multiple metabolically active enlarged lymph nodes throughout the body, with a maximum SUV of 4.2 in the right inguinal lymph node. A lymph node biopsy showed partial structural destruction, numerous scattered atypical cells, and mitotic figures, confirming lymphoma. The patient was diagnosed with SS (T4N3M0B2, stage IVA2) and was treated with methotrexate (15 mg/week), chidamide (30 mg twice a week), NB-UVB, and topical corticosteroids. After 1 year follow-up, the patient showed a resolution of generalized erythema and pustules, and new nail growth was observed.

### 2.3 Case 3

A 60-year-old man presented on 25 April 2024 with a 2-year history of rashes on the face, trunk, and extremities. Based on the Williams criteria, he was diagnosed with AD. The disease severity scores for the patient were as follows: SCORAD: 36, EASI: 13.7, PP-NRS: 7, DLQI: 12, and POEM: 13. After receiving five doses of dupilumab, his rash worsened. The patient had a history of allergic rhinitis. A specialist examination revealed dark red plaques of varying sizes with well-defined borders infiltrating the face, trunk, and extremities. Yellow-white scales were observed on plaques located on the buttocks, and scattered hyperpigmented patches were noted on the extremities (Figures 1G,H). Laboratory tests revealed no abnormalities. Skin pathology demonstrated focal hyperkeratosis, irregular epidermal hyperplasia, basal cell vacuolization, and lichenoid mononuclear cell infiltration in the superficial dermis. The infiltrating cells were slightly enlarged, anisotropic, and occasionally exhibited mitosis (Figure 2C). Immunohistochemical analysis revealed CD2 (+), CD3 (+), CD4 (+), CD5 (+), CD7 (partial+), CD8 (+), CD30 (approximately 10%), TCRB-C (+), and TCRG-A (+). PET/CT showed no significant abnormalities. The patient was diagnosed with MF (T2N0M0B0, stage IB). Dupilumab was discontinued, and treatment with curcumin (6 g/day), NB-UVB, and topical corticosteroids was initiated. After a 7-month follow-up, the infiltration of erythematous plaques was notably reduced.

To further investigate the relationship between biologic agents and CTCL, a literature review was conducted on 1 December 2024, using the PubMed, MEDLINE, and Embase databases. The primary search terms included “Atopic Dermatitis,” “Atopic Neurodermatitis,” “Atopic Eczema,” “Infantile Eczema,” “Upadacitinib,” “Dupilumab,” “Baricitinib,” “Abrocitinib,” “Tralokinumab,” “Delgocitinib,” “Lebrikizumab,” “Nemolizumab,” “Janus Kinase Inhibitors,” “JAK Inhibitors,” “JAKi,” “Biologics,” “Biological Products,” “Monoclonal Antibody,” “Monoclonal Antibodies,” “Cutaneous T-cell Lymphoma,” “Mycosis Fungoides,” and “Sezary Syndrome.” A total of 19 studies involving 31 patients (17 males and 14 females, mean age 55.48 ± 13.68 years) were identified (4–22). All patients were initially diagnosed with AD before receiving biologic agents, with 27 cases (87.10%) primarily treated with dupilumab. Among these 27 cases, there were 21 MF (77.78%), 2 SS (7.41%), 1 CTCL-NOS (3.70%), 1 CD30 + LPD (3.70%), 1 PCGD-TCL (3.70%), and 1 PTCL-NOS (3.70%). The most common stages were IIIA (16.13%) and IB (12.90%). Follow-up outcomes showed that 15 patients (48.39%) were alive with disease (AWD), 1 (3.23%) was alive without disease (AWOD), 4 (12.90%) had died of the disease (DOD), and 11 (35.48%) had unclear outcomes.

## 3 Discussion

AD is a benign, T-cell-mediated inflammatory skin condition, and biologic agents have become a cornerstone in its treatment. However, the early clinical and histopathological features of CTCL often mimic those of AD, making differentiation between the two conditions challenging. This similarity can lead to misdiagnosis, resulting in delayed treatment, increased healthcare costs, and potentially worse outcomes for patients (2). Furthermore, emerging studies suggest a possible association between biologic agents and CTCL, although it remains unclear whether CTCL is being misdiagnosed as AD or if biologic agents contribute to the progression of AD into CTCL (3). In this study, we report two cases of MF and one case of SS in patients who were initially diagnosed with AD and treated with biologic agents. Additionally, a comprehensive literature review highlights a significant association between biologic agents and CTCL, underscoring the need for further research to elucidate the underlying mechanisms (see Table 1).

**Table 1:** Demographic, clinical, and follow-up data of patients with biologic-associated cutaneous T-cell lymphoma.

No	Year	Author	Sex	Age	Clinical diagnosis	Pathology	Concomitant medications	Biological preparation	CTCL	Stage	Treatment	Outcome
1	2014	Jacks	F	35	Chronic dermatitis	Spongiotic dermatitis	Glucocorticoids	Adalimumab	PCAECTCL	N/A	Glucocorticoids	DOD
2	2018	Martinez-Escala	M	66	Eczema	N/A	MTX, NB-UVB, and PUVA	Infliximab	MF	IIIA	HDACi and Acitretin	AWD
3			M	75	Erythroderma	Spongiotic dermatitis	Glucocorticoids	Adalimumab	MF	IIIA	N/A	N/A
4	2019	Chiba	M	58	AD	N/A	Glucocorticoids	Dupilumab	MF	N/A	Glucocorticoids and NB-UVB	N/A
5	2020	Espinosa	M	64	AD	N/A	Glucocorticoids and AZA	Dupilumab	CTCL-NOS	N/A	Bexarotene	AWD
6			M	72	AD	N/A	MTX	Dupilumab	MF	IB	Glucocorticoids and NB-UVB	AWD
7			F	59	AD	N/A	Glucocorticoids and TCI	Dupilumab	MF	IA	Dupilumab	AWD
8			F	40	AD	N/A	Glucocorticoids	Dupilumab	MF	IIIA	Glucocorticoids, MTX, and NB-UVB	AWD
9	2020	Hollins	M	61	Eczema	eczema	Glucocorticoids and NB-UVB	Dupilumab	MF	N/A	N/A	N/A
10			F	60	Eczema	Eczema	Glucocorticoids and NB-UVB	Dupilumab,	MF	N/A	Glucocorticoids and NB-UVB	AWD
11	2020	Miyashiro	F	51	AD	spongiotic dermatitis	AZA	Dupilumab	MF	IIB	Acitretin and PUVA	AWD
12	2020	Tran	M	64	Erythroderma	N/A	Glucocorticoids	Dupilumab and guselkumab	SS	IVA1	Bexarotene	N/A
13	2020	Lazaridou	F	37	Erythroderma	Spongiotic dermatitis	Glucocorticoids, PUVA, TCI, MTX, and CsA	Dupilumab	SS	N/A	Mogamulizumab	AWD
14	2021	Sokumbi	F	66	Chronic dermatitis	Spongiotic dermatitis	Glucocorticoids, MTX, and MMF	Adalimumab and dupilumab	MF	IIA	N/A	AWD
15			F	65	AD	Spongiotic dermatitis	Glucocorticoids	Dupilumab	MF	IB	N/A	N/A
16			M	74	AD	Spongiotic dermatitis	Glucocorticoids and TCI	Dupilumab and apremilast	MF	IIIA	N/A	N/A
17			M	73	Chronic dermatitis	Spongiotic dermatitis	Glucocorticoids, MTX, CsA, MMF, and AZA	Omalizumab and dupilumab	MF	IIIA	N/A	AWD
18			M	74	Chronic dermatitis	Spongiotic dermatitis	Glucocorticoids, NB-UVB, MTX, MMF, and acitretin	Seukinumab and dupilumab	MF	IIA	N/A	N/A
19			F	44	AD	N/A	Glucocorticoids and MTX	Dupilumab	MF	IVA2	N/A	DOD
20			M	27	AD	N/A	Glucocorticoids and CsA	Dupilumab	MF	IB	N/A	AWD
21	2021	Ayasse	F	40	AD	Spongiotic dermatitis	MTX	Dupilumab	MF	IIB	N/A	AWD
22	2021	Du-Thanh	F	50	AD	Spongiotic dermatitis	Glucocorticoids, CsA, and MTX	Dupilumab	CD30 + LPD	N/A	Brentuximab Vedotin	DOD
23	2021	Newsom	F	48	AD	Spongiotic dermatitis	Glucocorticoids, PUVA, and MTX	Dupilumab	MF	IB	N/A	N/A
24			M	55	AD	N/A	Glucocorticoids and PUVA	Dupilumab	MF	IB	N/A	N/A
25	2021	Russomanno	M	43	AD	Spongiotic dermatitis	Glucocorticoids	Dupilumab	MF	IVA2	Brentuximab Vedotin	AWD
26	2022	Ahatov	F	62	AD	N/A	None	Dupilumab	PCGD-TCL	N/A	Glucocorticoids,	DOD
27	2022	Nakazaki	M	47	AD	N/A	None	Dupilumab	PTCL-NOS	N/A	Brentuximab Vedotin	AWD
28	2022	Park	M	72	AD	Spongiotic dermatitis	Glucocorticoids and TCI	Dupilumab	MF	IIA	Bexarotene	AWD
29	2023	Hsieh	M	34	AD	Spongiotic dermatitis	AZA and CsA	Baricitinib and dupilumab	MF	IIIA	Brentuximab Vedotin	AWOD
30	2023	Malick	F	60	AD	N/A	N/A	Dupilumab	MF	N/A	Acitretin, ECP	N/A
31	2024	Mo	M	44	AD	N/A	Glucocorticoids	Upadacitinib	MF	IVA1	MTX	N/A
32	2025	This study	F	39	AD	N/A	Glucocorticoids	Dupilumab, baricitinib, and upadacitinib	MF	IIA	Glucocorticoids, Acitretin, MTX, and NB-UVB	AWD
33			F	40	Erythroderma	N/A	Glucocorticoids and Acitretin	Dupilumab and upadacitinib	SS	IVA2	Glucocorticoids, MTX, HDACi, and NB-UVB	AWD
34			M	60	AD	N/A	Glucocorticoids, TCI	Dupilumab	MF	IB	Glucocorticoids and NB-UVB	AWD

AWD: alive with disease; AWOD: alive without disease; CD30 + LPD: CD30 + T-cell lymphoproliferative disorder; CSA: cyclosporin A; CTCL-NOS: cutaneous T-cell lymphoma not otherwise specified; DOD: dead of disease; ECP: extracorporeal photopheresis; HDACi: histone deacetylase inhibitor; IFN: interferon; MF: mycosis fungoides; MMF: mycophenolate mofetil; MTX: methotrexate; N/A: not available or not applicable; NB-UVB: narrowband ultraviolet B; PCAECTCL: primary cutaneous aggressive epidermotropic cytotoxic T-cell lymphoma; PCGD-TCL: primary cutaneous gamma-delta T-cell lymphoma; PTCL-NOS: peripheral T-cell lymphoma, not otherwise specified; PUVA: psoralen plus ultraviolet A; SS: Sézary syndrome; TCI: topical calcineurin inhibitor.

Dupilumab, an IL-4Rα-targeted biologic agent, works by modulating IL-4 and IL-13 signaling to suppress inflammation (23). However, studies have shown that patients with CTCL misdiagnosed as AD often experience worsening skin lesions following treatment with dupilumab. Furthermore, research indicates that even AD patients without prior misdiagnosis may have an increased risk of developing CTCL after receiving dupilumab, suggesting a potential role for this agent in CTCL onset (3). Notably, skin lesions in these cases often improve after discontinuing dupilumab. This phenomenon may be explained by dupilumab’s inhibition of IL-13Rα1, which leads to the upregulation of IL-13Rα2, a receptor associated with tumorigenesis. Activation of IL-13R*α*2 can stimulate cyclin-D1, promoting cell proliferation and potentially contributing to the development of CTCL (24). In our study, all cases had been treated with dupilumab, consistent with findings from prior research. These observations suggest that dupilumab may play a role in the onset and progression of CTCL.

Other biologic agents have also been implicated in CTCL. For instance, TNF-α inhibitors (adalimumab, etanercept, and infliximab) and IL-12/IL-23 inhibitors (ustekinumab and guselkumab) have been linked to CTCL development in various studies (3). Similarly, Janus kinase inhibitors (JAKIs), another class of biologic agents commonly used for AD, may also contribute to CTCL. Kolkowski et al. reported an increased risk of CTCL in patients treated with ruxolitinib (25), while Mo et al. suggested that upadacitinib might induce CTCL, potentially due to the dysregulation of the JAK3-STAT3 signaling pathway, which is associated with CTCL (22). In our study, two cases of MF received upadacitinib. However, the exact relationship between JAKIs and CTCL remains unclear and warrants further investigation.

Given the lack of robust evidence and a clear mechanism, the observed association between biologic agents and CTCL cannot rule out the possibility that CTCL is being misdiagnosed as AD. The early manifestations of CTCL closely resemble those of AD, and cases of misdiagnosed CTCL have been reported by Umemoto et al. (26) and Chiba et al. (6). Avoiding such diagnostic errors is crucial for optimizing clinical decision-making and further research. Our study, which documents all cases associated with biologic agents, is the first of its kind in China, filling a critical knowledge gap in this emerging clinical phenomenon. Based on our findings, we recommend that, when AD patients present with atypical skin lesions, demonstrate a poor response to respond to biologic agents, or experience paradoxical skin symptoms beyond exacerbation during treatment, an immediate skin biopsy should be performed to confirm or exclude CTCL. This is especially relevant for older patients without a history of allergic conditions. This approach can improve dermatologists’ awareness of such cases, facilitate earlier diagnosis and treatment, and potentially lead to better patient outcomes. Additionally, these findings may help reduce unnecessary therapeutic trials and resource utilization, ultimately creating substantial benefits for both patients and healthcare systems.

Our study has several limitations. First, the sample size was small, which may limit the generalizability of our findings. Second, the retrospective nature of the study lacked a control group, which could have provided more robust comparative data. Finally, the literature review may have been influenced by information bias. Despite these limitations, we emphasize that the clinical presentations of AD and CTCL are often strikingly similar, making differentiation difficult. When patients fail to achieve satisfactory results after receiving biologic agents, it is essential to thoroughly analyze their medical history, skin lesion characteristics, and treatment response. Performing a timely skin biopsy can help clarify the diagnosis and ensure appropriate management at the earliest possible stage.

## Data Availability

The original contributions presented in the study are included in the article/supplementary material, further inquiries can be directed to the corresponding author.
